# An Address on Insanity, and the Establishment of a Lunatic Asylum, Delivered before the Committee of the House of Representatives on Education, and the Public, Dec. 25, 1843

**Published:** 1845-04

**Authors:** 


*An Address on Insanity, and the establishment of a Lunatic Asylum*, delivered before the Committee of the House of Representatives on Education, and the public, December 25th, 1843, by JOHN EVANS, M. D., of Attica, Ind.

A notice of this Address may be found in the May No. of our 1st Vol. It has been republished in a pamphlet form, at the request of the Senate's Committee on Education, for the information of both houses of the Legislature of Indiana. In our former notice we expressed the hope that it might be widely circulated and extensively read.—ED.

